# Dietary Zinc Supplementation to Prevent Chronic Copper Poisoning in Sheep

**DOI:** 10.3390/ani8120227

**Published:** 2018-11-30

**Authors:** Antonio Humberto Hamad Minervino, Marta López-Alonso, Raimundo Alves Barrêto Júnior, Frederico Augusto Mazzocca Lopes Rodrigues, Carolina Akiko Sato Cabral Araújo, Rejane Santos Sousa, Clara Satsuk Mori, Marta Miranda, Francisco Leonardo Costa Oliveira, Alexandre Coutinho Antonelli, Enrico Lippi Ortolani

**Affiliations:** 1 Laboratory of Animal Health (LARSANA), Federal University of Western Pará (UFOPA), Rua Vera Paz, S/N, Salé, CEP 68040-255 Santarém, PA, Brazil; 2 Departamento de Patoloxía Animal, Facultade de Veterinaria, Universidade de Santiago de Compostela, 27002 Lugo, Spain; marta.lopez.alonso@usc.es; 3 Department of Animal Science, Federal Rural University of the Semiarid Region (UFERSA), Av. Francisco Mota, S/N, Bairro Pres. Costa e Silva, CEP 59625-900 Mossoró, RN, Brazil; barreto@ufersa.edu.br; 4 Department of Clinical Science, College of Veterinary Medicine and Animal Science, University of Sao Paulo (FMVZ/USP), Av. Prof. Orlando Marques de Paiva, 87, Cidade Universitária, CEP 05508-270, São Paulo, SP, Brazil; fmazzocca@usp.br (F.A.M.L.R.); mvcarolcabral@gmail.com (C.A.S.C.A.); rejane.santossousa@gmail.com (R.S.S.); clarasat@usp.br (C.S.M.); oliveiraflc@usp.br (F.L.C.O.); ortolani@usp.br (E.L.O.); 5 Departamento de Anatomía Produción Animal e Ciencias Clínicas Veterinarias, Facultade de Veterinaria, Universidad de Santiago de Compostela, 27002 Lugo, Spain; marta.miranda@usc.es; 6 Federal University of Vale do São Franciso (UNIVASF), Av. José de Sá Maniçoba, S/N, Centro, CEP: 56304-917, Petrolina, PE, Brazil; alexandre.antonelli@univasf.edu.br

**Keywords:** chronic copper poisoning, toxicity, sheep, liver, metallothionein

## Abstract

**Simple Summary:**

Sheep are susceptible to copper toxicosis, a deadly disease that usually occurs when the animals ingest large amounts of this mineral. Considering that the susceptibility of sheep to copper accumulation varies widely among breeds and from animal to animal., we evaluate whether Zn supplementation could be an option as a preventive measure to protect against hepatic Cu accumulation in sheep. Zn at 300 mg/kg dry matter (DM) is useful for preventing excessive hepatic Cu accumulation. Hepatic Cu accumulation is lower in animals receiving the Zn supplementation.

**Abstract:**

The aim of this study was to evaluate whether zinc (Zn) supplementation protects against hepatic copper (Cu) accumulation in copper-loaded sheep. Forty cross-bred lambs were assigned to five experimental groups. These included the control group (C) and four treatment groups that received Cu and/or Zn supplementation (dry matter (DM) basis) over 14 weeks, as follows: Cu (450 mg Cu/kg); Zn-35 (450 mg Cu + 35 mg Zn/kg); Zn-150 (450 mg Cu + 150 mg Zn/kg); and Zn-300 (450 mg Cu + 300 mg Zn/kg). Blood, liver, and bile samples were obtained for mineral determination by inductively coupled plasma optical emission spectrometry (ICP–OES). The hepatic metallothionein (MT) concentrations were also determined. At the end of the experiment, hepatic Cu concentrations were higher in all Cu-supplemented groups than in C. Hepatic Cu accumulation was lower in the groups receiving the Zn supplementation than in the Cu group, although the difference was only statistically significant (66%) in the Zn-300 group. The MT concentrations tended to be higher (almost two-fold) in the Zn groups (but were not dose related) than in the C and Cu groups, and they were related to hepatic Zn concentrations. Zn supplementation at 300 mg/kg DM is useful for preventing excessive hepatic Cu accumulation in sheep exposed to high dietary concentrations of Cu.

## 1. Introduction

Chronic copper poisoning (CCP) is a severe disease in sheep, causing the death of animals and economic losses worldwide [1,2]. The problem is particularly important in Brazil, where several outbreaks have been reported [3,4,5,6]. Primary CCP may be associated with ingestion of copper (Cu)-contaminated feedstuff [7], industrial waste, feeding broiler or pig manure [8], or the accidental use of mineral supplements containing high Cu levels that are formulated for other species [9,10]. Secondary CCP is also very common and appears to be associated with interactions in the gut with other essential trace elements such as sulphur (S), molybdenum (Mo), and iron (Fe) when these are not balanced in the diet [11,12]. As a result of these interactions, minimum requirements and maximum allowances of Cu are dependent on dietary Cu concentrations and are also difficult to establish.

Sheep appear to be very sensitive to CCP as, unlike most other species, they have a very limited capacity to excrete biliary Cu, as a result of a low capacity to accumulate Cu bound to metallothioneins (MT) in the liver. When Cu is absorbed from the intestine, it enters the liver, where it is utilized in normal hepatocyte metabolism; stored bound to MT; and, if the Cu balance is positive, excreted into the bile [13]. Metallothioneins appear to play a central role in Cu excretion into the bile, where Cu–MT is sequestered by the lysosomes for excretion [14]. If there is a large influx of Cu into the liver, the binding capacity of the MT and the removal of Cu–MT from the cytosol by lysosomes may be surpassed [13]. The Cu concentrations in cytoplasm thus increase, leading to lipid oxidation and oxidative damage in hepatocytes and erythrocytes and finally causing a haemolytic crisis [13,15]. Clinical signs in affected animals include hepatitis, anaemia, icterus, and haemoglobinuria [10,13,16,17].

Several therapies have been used to treat Cu-loaded or intoxicated animals, including different chelating agents [18] and molybdate therapies [19,20]. In areas where there is a high risk of CCP, Cu loading is mainly prevented by modifying dietary concentrations of Cu antagonists. Dietary supplementation with Mo and S has been useful in preventing the disease [12,21]. However, use of this measure in the field may be limited by the risk of inducing a Cu deficiency state or sulphur intoxication.

Recent studies have demonstrated that Zn therapy is safe in human patients suffering from Wilson’s disease [22,23] or dogs affected by chronic cumulative hepatopathologies [24,25]. Zinc probably protects against Cu toxicity in the liver by promoting the sequestration of free Cu in the non-toxic MT-bound form [26]. Although Cu can efficiently bind to MT in the liver, it is a weak MT inducer. By contrast, hepatic MT synthesis is highly regulated by Zn, and once Zn has induced MT synthesis, Cu can efficiently compete with Zn for MT-binding sites [13,27]. However, little is known about the usefulness of Zn for preventing or treating CCP in sheep, and the findings of some early studies seem contradictory. Thus, although the usefulness of Zn for protecting sheep against CCP has been demonstrated [28], no change in the hepatic Cu load was observed in sheep administered Zn therapy [29]. Moreover, breed differences have been described in sheep in relation to the interaction between Zn and Cu accumulation in the liver [30] and may be related to genetic differences in the capacity for MT to bind Cu and Zn.

The aim of this study was to evaluate whether different doses of dietary Zn protect against hepatic Cu accumulation in sheep exposed to high dietary concentrations of Cu. The effect of Zn on hepatic MT induction and its possible role in Cu biliary excretion were also investigated.

## 2. Material and Methods

### 2.1. Animals and Diet

Forty Santa Inês cross-bred male sheep (8 to 12 months old), of average weight 41.2 ± 4.1 kg, were used in the study. The animals were fed a basal diet of coast-cross (*Cynodon dactlylon*) hay (86.8% dry matter (DM), 32.4% crude fibre (CF), 7.8% crude protein (CP), 1.8% ether extract (EE), 6.3% ash) and commercial concentrate (87.0% DM, 16.0% CF, 14.0% CP, 2.0% EE, 16.0% ash), which represented 80% and 20% of the ration, respectively. The total amount of feed given was calculated for each individual sheep as 2.7% of body weight (DM basis) and was readjusted during the study. In addition, each animal received 12 g per day of a commercial mineral mixture (expressed in mg/kg Co: 40, Cu: 590, Fe: 1800, I: 80, Mn: 1300, Mo: 300, Zn: 3800) (Ovinofós, Tortuga Companhia Zootécnica Agrária, São Paulo, Brazil). The mineral supplement was formulated with sodium chloride, sulphur flower, dicalcium phosphate, sulphur transchelate, calcium iodate, manganese monoxide, cobalt transchelate, copper transchelate, manganese transchelate, selenium transchelate, zinc transchelate, cobalt sulphate, copper sulphate monohydrate, iron sulphate, zinc sulphate, and kaolin. Cu and Zn concentrations in the total diet were 15.2 and 95.0 mg/kg DM, respectively. Considering a sheep weighting 40 kg, the basal diet produced an intake of 12.8 mg Cu and 84.6 mg Zn per day.

The sheep were adapted to the experimental diet for 60 days prior to the study. They were kept in collective pens during the first 40 days of this adaptation period and then in metabolic cages (equipped with individual feeders and water suppliers) until the end of the study. The sheep were moved to a 25 m^2^ pen for 8–10 h once a week, to prevent the stress of being held in a metabolic cage for an extended period. The study was approved by the Bioethics Commission of the School of Veterinary Medicine, São Paulo University, protocol n. 1090/2017.

### 2.2. Experimental Design

The sheep were randomly assigned to five groups, each comprising eight individuals. The control group (C) received the basal diet with no additional supplements. Each of the four treatment groups received only Cu supplementation or supplementation with Cu plus different amounts of Zn, as follows: Cu (450 mg Cu/kg DM); Zn-35 (450 mg Cu + 35 mg Zn/kg DM); Zn-150 (450 mg Cu + 150 mg Zn/kg DM); and Zn-300 (450 mg Cu + 300 mg Zn/kg DM). Zinc oxide ACS (Synth, Diadema-SP, Brazil) was used as the Zn source and copper sulphate 5H_2_O ACS (Synth, Diadema-SP, Brazil) as the Cu source. The mineral supplements were prepared by adding the individual doses of Cu and Zn to the fixed amount of the commercial mineral premixture, before mixing with the amount of concentrate for each animal. The study lasted 14 weeks, after which all of the sheep were euthanized. The sheep were examined daily, and special attention was given to the presence of macroscopic haemoglobinuria.

### 2.3. Sampling

The sheep were weighed once a week, in the morning, before being fed. Blood samples were obtained at the beginning and the end of the study, by jugular venepuncture, and were stored in vacuum tubes with lithium heparin. Liver biopsies were performed on day 30 of the adaptation period using a surgical technique described by Minervino [31] and at the end of the study after slaughter (*postmortem* biopsy). Bile samples were collected at the same time. The liver and bile samples were stored at −80 °C until analysis.

### 2.4. Analytical Methods

Liver (0.5 g), bile (1.0 mL), and blood samples (1.0 mL) were acid-digested in an open system (4:1 nitric/perchloric acids; Synth, Diadema, Brazil) for Cu and Zn analysis [32]. Mineral concentrations were determined by inductively coupled plasma optical emission spectrometry (ICP–OES; Varian 710-ES, Varian Inc., Palo Alto, CA, USA). Analytical blanks and internal reference materials were run once every 20 samples, and recoveries ranged between 95 and 99%.

MT levels in the liver were determined using the cadmium saturation method [33]. Briefly, aliquots of 0.5 g of fresh liver were mixed in four volumes of sucrose solution (0.25 M, pH 8.4, 4 °C) with a homogenizing kit (TH115-PCR, Omni International Inc., Kennesaw, GA, EUA) and centrifuged (at 20,000× *g* for 20 min). The supernatant was saturated with 1 mL of CdCl_2_ solution (20 mg Cd/mL glycine buffer). Excess Cd was removed and precipitated by adding 0.2 mL of 2% haemoglobin solution (in buffer), before the mixture was heat-treated in a water bath (100 °C/1 min) and centrifuged at 1000× *g* for 5 min at room temperature. The final supernatant fraction was analyzed for Cd by ICP–OES, and the MT concentrations were calculated by assuming a Cd/MT molar ratio of 17. For quality control, blanks and internal reference materials were included, and the recovery rate was 96.7%.

### 2.5. Statistical Analysis

All statistical tests were conducted using SPSS (v.20.0). A Kolmogorov–Smirnov test was used to check the normality of the data distribution. One-way analysis of variance (ANOVA) and post hoc Tukey’s tests were used to test the influence of the treatments on Cu and Zn concentrations in blood, liver, and bile. The Pearson’s correlation coefficient was used to determine the correlations between Cu and Zn concentrations in the different tissues and MT concentrations in the liver. The study was approved by the Bioethics commission of the School of Veterinary Medicine, São Paulo University, protocol n. 1090/2017.

## 3. Results

### 3.1. Clinical Aspects and Mortality

The sheep in the C and Zn-300 groups did not show any clinical signs suggestive of CCP. However, one sheep in each of the Cu (at week 14), Zn-35 (at week 8), and Zn-150 (at week 10) groups developed the disease and had to be euthanized. In all cases, approximately two weeks before the acute haemolytic crisis, the sheep showed signs of hypoxaemia and weight loss (an average decrease in body weight of 22%). These symptoms were followed by macroscopic haemoglobinuria, severe apathy, yellowish mucus, increased heart rate and body temperature, reduced respiratory rate and ruminal movement, anorexia, and oliguria. Macroscopic features of CCP, including weight loss, yellowish serous fluid, orange-yellowish liver, blackened kidneys, and urine with haemoglobinuria, were observed at necropsy. No statistically significant effect of treatments on the frequency of mortality was observed (Fisher test, *p* = 0.12).

### 3.2. Cu and Zn Concentrations

The Cu and Zn concentrations in blood, liver, and bile in the five experimental groups, before and at the end of the experiment, are summarized in Table 1. Prior to the study, Cu concentrations in blood were in the marginal range (Cu: 0.3–0.6 mg/L, [34], and the Zn concentrations were normal. The hepatic concentrations (the best indicator of both element status) were adequate (Cu: 25–100 mg/kg fresh weight; Zn: 30–75 mg/kg, [35]. There were no statistically significant differences in the concentrations of any of the elements between groups. At the end of the experiment, blood Cu and Zn concentrations were within the adequate range in all sheep. The blood Cu concentrations were statistically significantly higher in the groups receiving Cu supplementation only than in the C group, but did not differ between each other, independently of the Zn dose. There were no statistically significant differences in blood Zn concentrations between groups in relation to the amount of Zn ingested, including the C and Cu groups receiving the basal diet.

Considering hepatic Cu accumulation at the end of the study, the Cu concentrations were significantly higher in the Cu treated groups, except the Zn-300 group, than in the C group. Hepatic Cu accumulation was lower in the groups receiving Zn supplementation than in the Cu group, although the difference was only statistically significant (66%) for the Zn-300 group. Hepatic Zn concentrations similarly increased in all the treated groups during the study, irrespective of the Zn dose (ca. 1.5-fold, including the Cu group), but decreased in the C group.

The Cu and Zn concentrations in the bile did not differ between groups prior to the study. At the end of the study, biliary excretion of Cu was higher (ca. six-fold) in the groups receiving the Cu treatment than in the C group, irrespective of the level of Zn administration. There were no differences between groups in Zn biliary excretion at the end of the experiment.

**Figure 1:**
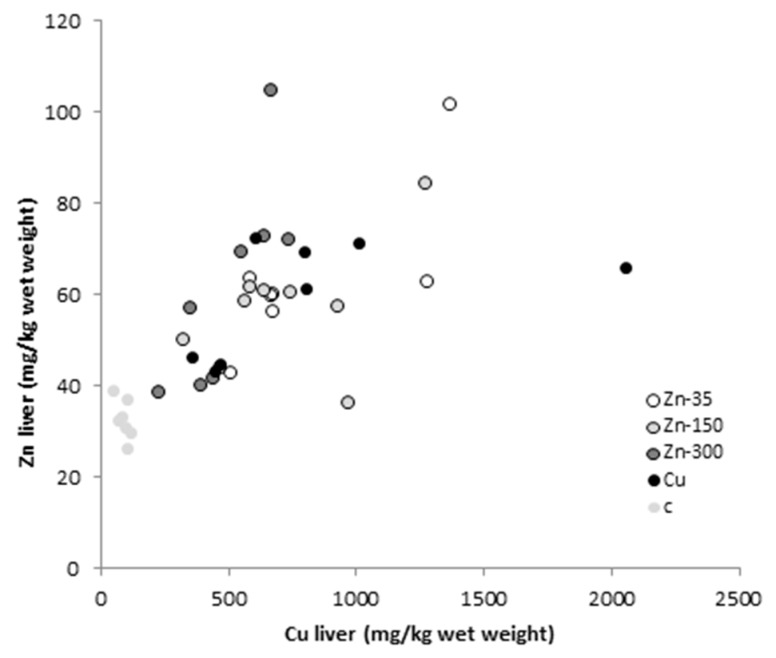
Scatter plot showing the relationship between Cu and Zn concentrations (mg/kg fresh weight) in the liver of sheep at the end of the study, for the five experimental groups: C, control (no treatment); Cu, 450 mg Cu/kg dry matter (DM); Zn-35, 450 mg Cu + 35 mg Zn/kg DM; Zn-150, 450 mg Cu + 150 mg Zn/kg DM; and Zn-300, 450 mg Cu + 300 mg Zn/kg DM.

There were no significant associations between Cu and Zn concentrations in the liver and those in the blood and bile either before or at the end of the experiment (*p* > 0.05 in all cases). By contrast, at the end of the study, the hepatic concentrations of Cu and Zn were correlated (R = 0.663, *p* < 0.001, Figure 1).

### 3.3. Metallothionein Concentrations

**Figure 2:**
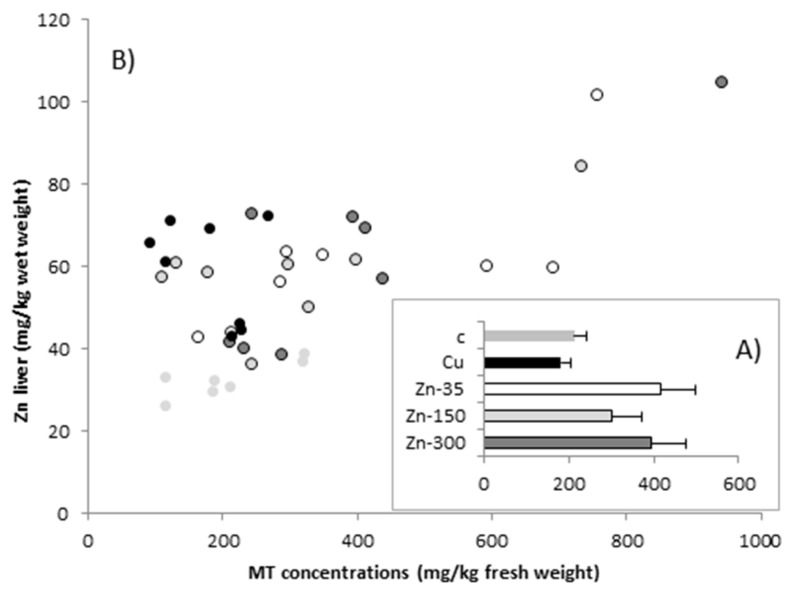
Scatter plot showing (**A**) metallothionein (MT) concentrations (expressed as mean ± SEM) and (**B**) the relationship between MT and Zn concentrations (mg/kg fresh weight) in the liver of sheep at the end of the study, for the five experimental groups: C, control (no treatment); Cu, 450 mg Cu/kg dry matter (DM); Zn-35, 450 mg Cu + 35 mg Zn/kg DM; Zn-150, 450 mg Cu + 150 mg Zn/kg DM; and Zn-300, 450 mg Cu + 300 mg Zn/kg DM.

At the end of the study, hepatic MT concentrations ranged from 92 to 940 mg/kg of fresh weight (Figure 2). Although the average MT concentrations were higher (nearly two-fold) in the groups receiving the Zn treatment than in the C and Cu groups, the differences were not statistically significant. Hepatic MT concentrations were not correlated with Cu concentration in the liver (*p* > 0.05), but were related to hepatic Zn concentrations (R^2^ = 0.383, F_(1,39)_ = 23.613, *p* < 0.001). The relationship can be expressed by the equation y = 1.957x − 65.05, where y is the liver MT concentration (mg/kg fresh weight) and x is the liver Zn concentration (mg/kg fresh weight).

## 4. Discussion

The study findings indicate that Zn supplementation at 300 mg/kg DM, a concentration well-tolerated by sheep [36,37], is useful for preventing excessive hepatic Cu accumulation in sheep exposed to high dietary concentrations of Cu.

It is known that (i) the marked inter-species differences in terms of susceptibility to hepatic Cu toxicity is related to the ability to synthesize hepatic MT [38,39], and (ii) Zn is a potent inductor of MT in the liver with hepatic MT concentrations being strongly related to the hepatic Zn [27]. However, hepatic MT induction does not seem to be the primary mechanism by which Zn exerted protection against hepatic Cu accumulation in the present study. The MT concentrations were closely correlated with Zn levels in the liver, but they did not increase proportionally in the Zn-dosed groups. Moreover, there were no differences in biliary Cu excretion between the Cu, Zn-35, Zn-150, and Zn-300 groups at the end of the study, also indicating that the lower hepatic Cu accumulation in the sheep receiving Zn supplementation was not related to higher Cu biliary excretion of the MT-bound Cu. Similar results have previously been described in Cu-loaded sheep [40], which were apparently not able to increase biliary Cu excretion in response to increased Cu intake.

Conversely, our findings seem to indicate that the protective effect of Zn in decreasing Cu loading may be related to the ability of Zn to decrease Cu absorption at the intestinal level. Zinc homeostasis is tightly regulated through intestinal Zn absorption, in which MT, in conjunction with other Zn transporters, plays a leading role [41,42]. Dietary restriction of Zn results in diminished MT synthesis and allows absorption of dietary Zn; however, above a certain threshold, MT synthesis increases and prevents excessive Zn absorption. This saturable mechanism explains why there were no significant differences in blood Zn concentrations between animals receiving the Zn-balanced basal diet (C and Cu groups) and those receiving the different Zn doses. Although Cu cannot synthesize MT in the gut, it effectively competes with Zn for the binding positions, resulting in low Cu absorption and, consequently, in secondary Cu deficiency in animals exposed to high dietary levels of Zn [43]. In this context, the competitive interaction may be a useful means of reducing Cu absorption in animals exposed to high levels of Cu.

Assuming that the protective effect exerted by Zn on hepatic Cu loading in our study occurred via modulation of Cu gut absorption, a negative association between the Zn dose and the Cu concentrations in the blood would be expected. However, Cu absorbed from the gut (Cu in albumin- and amino acid-bound forms) represents only a small proportion of the total concentration of Cu in the blood (5–10%) [43]. After adequate Cu status is reached, the activities of ceruloplasmin (corresponding to Cu from the liver exported to tissues) and Cu-dependent enzymes (such as superoxide dismutase in erythrocytes) reach maximal levels that are not increased on further hepatic accumulation of Cu [44]. In addition to dietary Cu, ceruloplasmin levels vary with factors such as age and sex [45,46], and rapidly increase in response to exercise and several inflammatory conditions and infection [36]. As a result of all of these factors, the blood concentration of Cu is a poor indicator of hepatic Cu loading [47,48]. Indeed, animals with deficient or almost deficient levels of Cu in blood were intoxicated or almost intoxicated, as indicated by necropsy and hepatic biopsy results [49]. Acute hepatic necrosis only develops when the liver stores reach a critical threshold, leading to release of Cu from the liver and causing transiently high serum Cu concentrations [50].

As already stated, after Cu leaves the portal circulation and reaches the liver, it is utilized in normal hepatocyte metabolism; stored bound to MT; and, if the Cu balance is positive, excreted into the bile bound to MT [13]. The capacity of the ovine liver to synthesize MT, and consequently to bind Cu, appears insufficient. Cu binding to MT was not assessed in the present study. However, considering that (i) each MT molecule binds 7 Cu ions [51], and that (ii) at high Cu liver storages nearly all positions were occupied by Cu [27], the amount of MT-bound Cu observed in the present study would represent less than 5% of the total Cu in the liver (considering a mean hepatic MT concentration of 400 mg/kg of fresh weight and a total Cu concentration of 600 mg/kg of fresh weight). Similar results have been described both in sheep [52] and in cattle [27] exposed to high dietary concentrations of Cu.

Finally, considering that in the present study blood Zn concentrations were not related to Zn supplementation (both control and Zn-dosed diets), the fact that hepatic Zn concentration was statistically significantly higher (two-fold) in all groups receiving Cu supplementation than in the control group is striking. Moreover, total hepatic Cu and Zn concentrations were correlated in all animals (Figure 1). As Cu and Zn are chemically similar, a competitive interaction has frequently been described [30,38], including a decrease in Cu intestinal absorption in Zn-dosed animals in the present study. However, the interaction between both elements in the liver was positive in our study and did not seem to be associated with the proportion of these elements bound to MT. A similar increase in hepatic Zn has been described in patients with Wilson’s disease [53] and may be related to oxidative damage caused by Cu loading. At high concentrations, Cu is not safely bound and acts as a pro-oxidant, promoting free radical damage and leading to an increase in Cu–Zn superoxide dismutase activity in the liver [54]. Indeed, increased superoxide dismutase concentrations have been described in Cu-loaded rats [55].

## 5. Conclusions

The study findings indicate that Zn supplementation at 300 mg/kg DM, a concentration well tolerated by sheep, is useful for preventing excessive hepatic Cu accumulation in sheep exposed to high dietary concentrations of Cu. Although intestinal MT concentrations were not measured in our study, the Cu and Zn concentrations in blood, liver, and bile in the experimental groups appear to indicate that the mechanism by which Zn exerts its protection is related to the effective induction of MT in the intestine, limiting intestinal Cu absorption and, consequently, the hepatic Cu loading. However, the low capacity of hepatic MT induction in sheep prevents Zn from producing (at least under the study conditions) an increase in biliary Cu excretion, as previously demonstrated in other animals. Further studies that assess the capacity of Zn to induce intestinal MT are needed to confirm our hypothesis and to determine the dose at which Zn supplementation exerts a protective effect against Cu intestinal absorption.

## Figures and Tables

**Table 1:** Cu and Zn mean concentrations in blood (mg/L), liver (mg/kg fresh weight), and bile (mg/L) in the experimental groups before and at the end of the study.

Experimental Groups *							
	C	Cu	Zn-35	Zn-150	Zn-300	RMSE	*p*
*Blood*							
Cu _*prior experiment*_	0.477	0.540	0.614	0.604	0.586	0.118	0.187
Cu _*end experiment*_	0.592 ^a^	0.827 ^b^	0.856 ^b^	0.855 ^b^	0.948 ^b^	0.319	0.001
Zn _*prior experiment*_	1.167	1.323	1.297	1.461	1.125	0.232	0.075
Zn _*end experiment*_	2.650	2.553	2.570	2.718	2.438	0.52	0.867
*Liver*							
Cu _*prior experiment*_	52.5	58.9	61.3	61.2	66.0	31.3	0.946
Cu _*end experiment*_	88.0 ^a^	817 ^b^	769 ^b^	746 ^b^	492 ^a^	332	0.001
Zn _*prior experiment*_	47.7	39.3	44.5	38.3	44.7	10.4	0.370
Zn _*end experiment*_	32.6 ^a^	59.3 ^b^	61.7 ^b^	59.2 ^b^	62.4 ^b^	15.7	0.004
*Bile*							
Cu _*prior experiment*_	0.217	0.117	0.194	0.146	0.102	0.118	0.384
Cu _*end experiment*_	0.350 ^a^	2.088 ^b^	2.225 ^b^	2.138 ^b^	1.938 ^b^	1.39	0.017
Zn _*prior experiment*_	1.055	0.578	0.714	0.442	0.457	0.602	0.307
Zn _*end experiment*_	1.381	1.003	0.891	0.977	0.920	0.564	0.432

* Experimental groups: C: control group; Cu: 450 mg Cu/kg dry matter (DM); Zn-35: 450 mg Cu + 35 mg Zn/kg DM; Zn-150: 450 mg Cu + 150 mg Zn/kg DM; Zn-300: 450 mg Cu + 300 mg Zn/kg DM. Lowercase letter in the same line indicate statistical difference between groups.. RMSE: root mean square error.
